# Utilization and Outcomes of Septuagenarian Donation After Circulatory Death Donors

**DOI:** 10.1097/TXD.0000000000002014

**Published:** 2026-09-25

**Authors:** Michael McCabe, Abigail Loszko, Justin Weissberg, Amit Nair, Nicholas Lim, Jeremy Taylor, Randeep Kashyap, Juliet Emamaullee, Koji Tomiyama, Roberto Hernandez-Alejandro, Yuki Bekki

**Affiliations:** 1 Transplant Institute, University of Rochester Medical Center, Rochester, NY.

## Abstract

**Background.:**

The ongoing organ shortage and aging global population necessitate the utilization of older donors. However, the transplantability of donation after circulatory death (DCD) grafts from septuagenarian donors remains uncertain.

**Methods.:**

Adult DCD donors in the United Network for Organ Sharing database between 2020 and 2024 were stratified on the basis of age 70 y or older (septuagenarian donors) and younger than 70 y. The utilization and posttransplant outcomes were analyzed.

**Results.:**

There were 24 258 DCD donors, of whom 195 (0.8%) were septuagenarians. Among 364 recovered kidneys, 58 livers, and 8 lungs from septuagenarian donors, 61 (16.7%) kidneys, 50 (86.2%) livers, and 6 (75%) lungs were transplanted. There was no significant difference in 1-y graft survival for either kidney (92.9 versus 91.6%, *P* = 0.99) or liver (92.3 versus 90.1%, *P* = 0.82) recipients. There was heterogeneity across regions in septuagenarian donor practices: 2 (3.6%) of 55 organ procurement organizations recovered 33.8% of all septuagenarian donors, whereas 4 (1.8%) of 218 centers transplanted 34.8% of all septuagenarian grafts used.

**Conclusions.:**

Septuagenarian DCD grafts achieved favorable graft survival after kidney or liver transplantation. Although significant variation exists across organ procurement organizations and transplant centers, aggressive exploration of septuagenarian DCD donors is warranted to resolve the organ shortage.

## INTRODUCTION

Donation after circulatory death (DCD) in the United States has increased markedly, more than doubling during a decade from 14.6% to 36.0% of all donors.^1^ In parallel, record-high numbers of kidney,^2^ liver,^3^ and lung^4^ transplants have been achieved, each driven by the expansion of deceased donation. Yet, waitlist numbers have reached all-time highs across organs, and waitlist mortality per 100 patient-years has remained high at 5.0 for kidney, 12.9 for liver, and 13.3 for lung.^2-4^ Expanding the deceased donor pool, therefore, remains critical.^5^

An aging global population, and thereby an aging donor pool, is a crucial factor influencing the availability of organ donors. The utilization of older donors has increased minimally in the last decade,^1^ yet individuals aged 70 y or older already comprise 12.3% of the national population, a share that will only increase.^6^ Aging is particularly relevant to DCD donors, because stringent age cutoffs have typically been applied to their utilization.^7-9^ However, age cutoffs have been gradually expanded in prior years, and sexagenarian DCD donors are now reported to achieve satisfactory posttransplant outcomes in kidney,^10^ liver,^11^ and lung^12^ transplants. Given that DCD transplant outcomes are close to achieving parity with donation after brain death (DBD),^13-15^ and with septuagenarian DBD grafts showing acceptable outcomes in kidney,^16^ liver,^17^ and lung transplant,^18^ grafts from septuagenarian DCD donors warrant evaluation. However, to date, no study has examined either the utilization or outcomes of septuagenarian DCD donors using US national data.

Concurrently, significant technological advances in organ perfusion changed the landscape of transplantation. Normothermic regional perfusion (NRP), adopted in the United States in 2020, has been shown to improve both utilization and posttransplant outcomes for kidney and liver grafts.^19^ Ex situ machine perfusion (es-MP) for liver grafts, which received Food and Drug Administration approval in 2021,^20^ and lung es-MP, approved in 2019,^21^ have both also been shown to substantially increase organ utilization. With improved ischemic recovery and enhanced abilities for organ diagnostics and therapeutics, it is expected that these technologies will further augment the use of DCD and “risker” grafts, including those from older donors.^19-22^ Therefore, the objective of this study was to determine the prevalence and trends in utilization of septuagenarian DCD allografts, along with their posttransplant outcomes in the United States. Furthermore, organ procurement organization (OPO) and transplant center-level analyses were performed to assess the impact of perfusion technologies on transplant practices using septuagenarian DCD donors.

## MATERIALS AND METHODS

The United Network for Organ Sharing (UNOS) Standard Transplant Analysis and Research database was used. Adult (age 18 y or older) DCD donors who underwent organ recovery between January 1, 2020, and December 31, 2024, were included. The study start year was chosen to correspond to the inflection point in the increasing utilization of NRP in the United States.^23^ Donors were categorized into septuagenarian (age 70 y or older) and non-septuagenarian (age younger than 70 y) cohorts; pertinent donor and recipient clinical and demographic characteristics were compared between the cohorts. The study was exempted from institutional review board approval as it did not qualify as human subject research, as only de-identified retrospective data were used. Informed consent was waived as all data were fully de-identified before transmission to the investigators.

Organ utilization rate was assessed as the number of recovered organs ultimately transplanted.^2-4^ Primary nonfunction (PNF) for kidney grafts was defined as a return to dialysis dependence or an estimated glomerular filtration rate of 20 mL/min/1.2 m^2^ within the first 90 d after the first week after transplant.^24^ PNF for liver grafts was defined as severe allograft dysfunction immediately following transplant, resulting in either retransplantation or death.^25^ PNF for lung grafts was defined as when graft failure had occurred, and “primary failure” was indicated as the cause in the database. Delayed graft function (DGF) was defined as when a kidney transplant recipient required dialysis within the first week of transplant.^26^ For liver donors, the donor risk index (DRI) was calculated according to Feng et al.^27^

Utilization of NRP was defined as a difference in death and clamp time ≥50 min.^28^ For evaluation of es-MP of liver grafts, to account for the known underreporting of es-MP in the UNOS database, livers were considered to have undergone es-MP either if (1) it was indicated as es-MP in the database or (2) if there was a preservation time of ≥10 h.^29^

To evaluate geographic variation in septuagenarian DCD utilization, septuagenarian donors and recipients of septuagenarian grafts were stratified by Organ Procurement and Transplantation Network (OPTN) region, OPO, and transplant center. The association between the use of NRP, liver es-MP, kidney es-MP, lung es-MP, and septuagenarian donor recovery per OPO was analyzed with Spearman’s rank correlation. Additionally, a supplementary analysis was conducted to determine the number of potential septuagenarian DCD donors. Aggressive OPOs were defined as those at or above the 90th percentile for proportion of septuagenarians recovered. The median proportion of septuagenarians recovered in the aggressive cohort was applied to the remaining OPOs to determine the number of additional donors that may have been recovered.

Statistical analysis was performed using R version 4.5.1.^30^ Data were summarized using median values and interquartile ranges for continuous variables and counts with percentages for categorical variables. Statistical significance was set at a *P *value of <0.05. Differences between donor cohorts were compared using the Wilcoxon rank-sum test for numeric/continuous variables, and the chi-square test for categorical variables. Graft survival was evaluated using the Kaplan-Meier method and compared between cohorts using the log-rank test. Graft survival for all organs was calculated from time of transplant until death, graft loss, or date of last follow-up. Individual models of graft survival were created, with survival truncated and right censored at 1 y.

The data reported here were supplied by UNOS as the contractor for the OPTN. The interpretation and reporting of these data are the sole responsibility of the study authors and should in no way be seen as an official policy or interpretation by the OPTN or the US Government.

## RESULTS

### Donor Cohort

During the study period, there were a total of 74 679 deceased organ donors in the United States. After excluding 49 310 DBD donors and 4493 pediatric donors, a cohort of 24 258 DCD donors remained. Of these, 195 (0.8%) were septuagenarian donors. Overall numbers of septuagenarian DCD donors increased dramatically from 2020 to 2024, with 3, 9, 20, 22, and 141 donors recovered, respectively. From 195 septuagenarian donors, 364 kidneys, 58 livers, and 8 lungs were recovered (Figure 1). There were no instances of heart, pancreas, or intestine recovery from septuagenarian donors during the study period. compared with non-septuagenarian donors, the rate of organ recovery was lower in septuagenarian donors for kidneys (93.8 versus 97.7%, *P* = 0.002), livers (29.7 versus 39.2%, *P* = 0.007), and lungs (2.1 versus 7.4%, *P* = 0.004). The utilization rate of kidneys from septuagenarian donors was significantly lower (18.6 versus 66.7%, *P* < 0.001). There was no significant difference in the rate of liver (86.2 versus 75.5%, *P* = 0.55) or lung (75.0 versus 70.5%, *P* > 0.9) utilization. Reasons for kidney and liver nonutilization are given in **Tables S1 and S2** (**SDC,** https://links.lww.com/TXD/A910), respectively.

**FIGURE 1.**
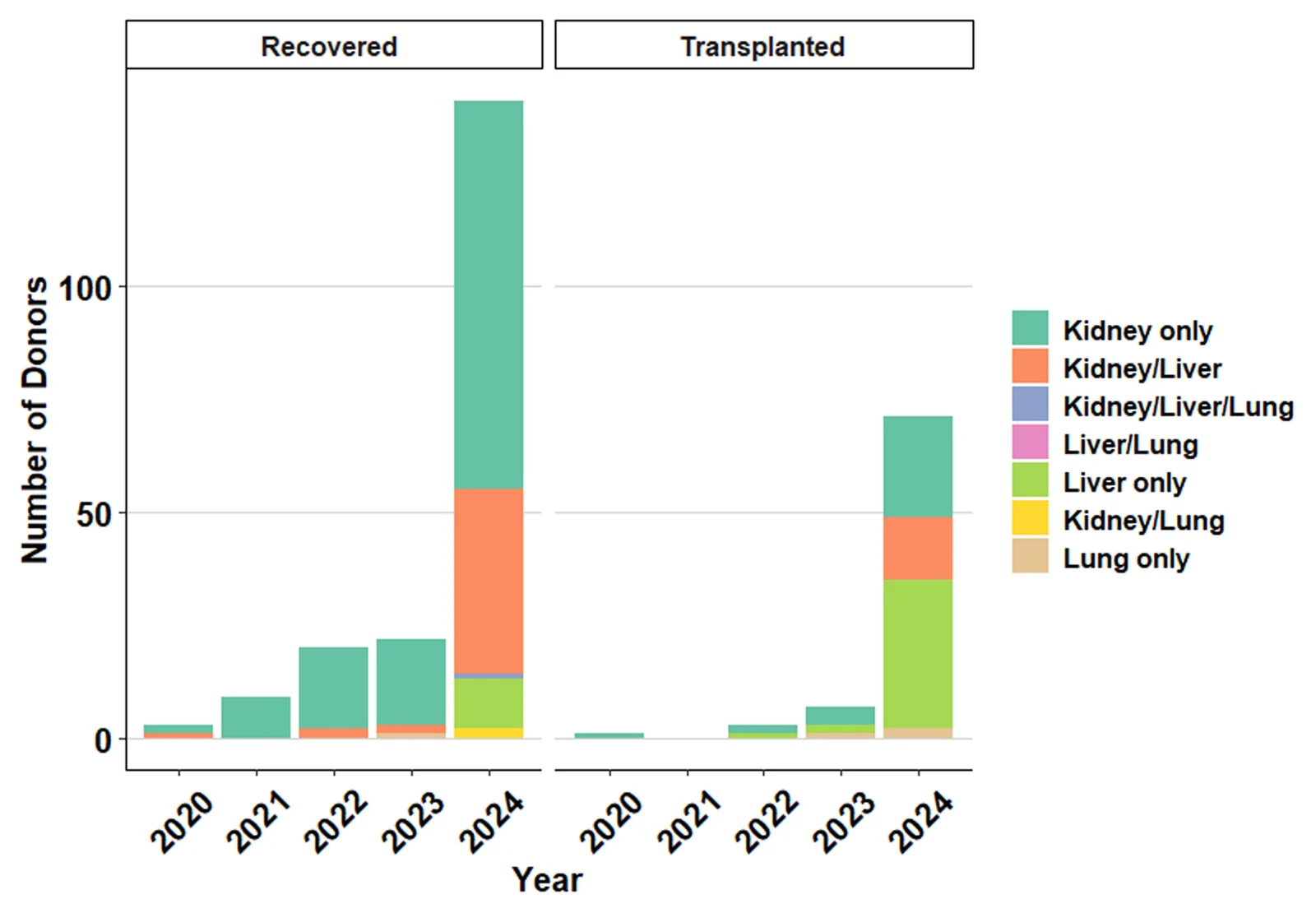
Total number of septuagenarian donation after circulatory death (DCD) donors recovered and transplanted by organ, 2020–2024.

Potential DCD donor demographics of the septuagenarian and non-septuagenarian cohorts are shown in Table 1. Al-though septuagenarian donors were significantly more likely to have died of stroke (33.3 versus 21.5%, *P *= 0.004), they were less likely to have died of drug overdose (1.0 versus 12.3%, *P* < 0.001). The DRI of septuagenarian donors was significantly higher (2.98 versus 2.01, *P *< 0.001). There was no significant difference in the prevalence of diabetes between cohorts (23.0 versus 18.4% *P* = 0.10). There was no significant difference in terminal serum total bilirubin (0.60 versus 0.60 mg/dL, *P* = 0.10), creatinine (0.80 versus 0.86 mg/dL, *P* = 0.06), or blood urea nitrogen (24 versus 22 mg/dL, *P* = 0.06), nor was there a significant difference in liver macrosteatosis (5 versus 5%, *P *= 0.8). Septuagenarian DCD donors were significantly more likely to undergo NRP than non-septuagenarian donors (32.8 versus 8.5%, *P* < 0.001), as well as for liver machine perfusion to be used (67.2 versus 42.9%, *P* < 0.001). There was no difference in the utilization of kidney es-MP between the septuagenarian and non-septuagenarian cohorts (82.9 versus 78.0%, *P* = 0.14). Of recovered lungs, 50.0% (2) of the septuagenarian cohort underwent es-MP, compared with 42.4% (757) of the non-septuagenarian cohort (*P *= 0.60).

**TABLE 1.** Characteristics of potential DCD donors

	Septuagenarian DCD (N = 195)	Non-septuagenarian DCD (N = 24 064)	*P* ^*a*^
Age, y	71 (70–73)	50 (38–58)	<0.001
Sex, M	101 (51.7%)	15 654 (65.0%)	<0.001
Race			0.078
White	164 (84.1%)	18 538 (77.0%)	
Black	12 (6.2%)	2262 (9.4%)	
Hispanic/Latino	11 (5.6%)	2431 (10.1%)	
Asian	4 (2.1%)	458 (1.9%)	
Other	4 (2.1%)	375 (1.6%)	
BMI, kg/m^2^	28 (24–34)	29 (25–34)	0.13
Cause of death			0.004
Anoxia	90 (46.1%)	12 512 (51.9%)	
Head trauma	30 (15.3%)	4331 (17.9%)	
Stroke	65 (33.3%)	5182 (21.5%)	
CNS tumor	0 (0%)	39 (0.2%)	
Other	10 (5.1%)	1999 (8.3%)	
Mechanism of death			<0.001
Asphyxiation	12 (6.2%)	1132 (4.7%)	
Cardiovascular	54 (27.6%)	6515 (27.0%)	
Drug intoxication	2 (1.0%)	2972 (12.3%)	
Trauma	127 (65.1%)	13 445 (55.8%)	
History of diabetes	45 (23.0%)	4449 (18.4%)	0.10
History of hypertension	130 (66.6%)	11 046 (45.9%)	<0.001
Terminal ALT, U/L	30 (19–56)	41 (23–84)	<0.001
Terminal AST, U/L	46 (28–74)	53 (32–97)	<0.001
Terminal bilirubin, mg/dL	0.60 (0.40–0.80)	0.60 (0.40–1.00)	0.10
Terminal creatinine, mg/dL	0.80 (0.63–1.15)	0.86 (0.61–1.40)	0.055
Terminal BUN, mg/dL	24 (16–36)	22 (15–34)	0.063
NRP used	64 (32.8%)	2048 (8.5%)	<0.001
Time to death,^*b*^ min	23.0 (17.0–36.0)	20.0 (14.0–28.0)	<0.001
Total WIT,^*c*^ min	30.0 (24.0–45.0)	27.0 (22.0–37.0)	<0.001
Liver es-MP used	39 (67.2%)	4057 (42.9%)	<0.001
Kidney es-MP used	151 (82.9%)	18 345 (78.0%)	0.14
Lung es-MP used	1 (0.5%)	330 (1.4%)	0.5
Kidney biopsy obtained	180 (92.3%)	17 529 (72.8%)	<0.001
Liver biopsy obtained	37 (18.9%)	4704 (19.5%)	0.14
Liver macrosteatosis, %	5 (0–10)	5 (0–10)	0.8

Data presented as median (IQR) or n (%).

^*a*^Wilcoxon rank-sum test; Pearson’s chi-square test; Fisher exact test.

^*b*^Time from withdrawal to declaration of death, calculated for all donors. Missing in 3.5% septuagenarian; 8.2% of non-septuagenarian.

^*c*^Time from withdrawal to cold flush, calculated for non-NRP donors. Missing in 0.6% of non-septuagenarian donors.

ALT, alanine aminotransferase; AST, aspartate aminotransferase; BMI, body mass index; BUN, blood urea nitrogen; CNS, central nervous system; DCD, donation after circulatory death; es-MP, ex situ machine perfusion; IQR, interquartile range; NRP, normothermic regional perfusion; WIT, warm ischemia time.

### Characteristics of Kidney Donors and Recipients

Recipients of kidneys from septuagenarian donors were significantly older than recipients of kidneys from non-septuagenarian donors (69.0 versus 58.0 y, *P* < 0.001; Table 2). They were also significantly more likely to undergo preemptive transplant (32.7 versus 18.2%, *P* = 0.02), and if they were on dialysis, they spent significantly less time on dialysis (2.63 versus 3.94 y, *P* < 0.001). There was a significantly increased prevalence of diabetes in recipients of septuagenarian kidneys (62.2 versus 44.4%, *P* = 0.005). Transplanted kidneys from septuagenarian donors were significantly more likely to have been on es-MP than the non-septuagenarian cohort (96.7 versus 82.9%, *P* = 0.005), and to have come from a donor where NRP was used (34.4 versus 9.2%, *P* < 0.001). Recipients of septuagenarian kidneys were significantly more likely to receive a dual kidney transplant than standard age recipients (11.4 versus 0.9%, *P* < 0.001).

**TABLE 2.** Demographic and clinical characteristics of kidney recipients

	Septuagenarian DCD (N = 61)	Non-septuagenarian DCD (N = 30 944)	*P* ^*a*^
Recipient			
Age, y	69 (65–73)	58 (49–66)	<0.001
Sex, male	34 (55.7%)	19 060 (61.5%)	0.3
BMI, kg/m^2^	28.8 (26.5–32.3)	28.3 (24.7–32.4)	0.4
Recipient history of diabetes	38 (62.2%)	13 755 (44.4%)	0.005
EPTS	0.76 (0.66–0.95)	0.57 (0.30–0.81)	<0.001
Previous kidney transplant	2 (3.3%)	2712 (8.8%)	0.2
On dialysis at transplant	41 (67.2%)	25 274 (81.6%)	0.021
Time on dialysis, y	2.63 (1.56–3.48)	3.94 (2.17–5.98)	<0.001
Waitlist time, d	248 (94–860)	376 (84–1081)	0.14
Donor			
Age, y	70 (70–72)	45 (34–54)	<0.001
Terminal serum creatinine, mg/dL	0.67 (0.55–0.82)	0.80 (0.60–1.15)	0.001
KDPI	0.91 (0.84–0.95)	0.55 (0.37–0.73)	<0.001
HLA mismatch			>0.9
0	2 (3.3%)	1474 (4.8%)	
1–2	59 (96.7%)	29 468 (95.2%)	
Transplant			
Kidney graft type			<0.001
Single kidney	54 (88.5%)	30 539 (98.6%)	
Dual kidney	7 (11.4%)	281 (0.9%)	
En bloc	0 (0%)	8 (<0.1%)	
Preservation time, h	20.7 (17.7–24.5)	20.4 (16.6–24.4)	0.4
Distance from transplant center, nautical miles	128 (62–208)	108 (27–204)	0.2
es-MP used	59 (97%)	25 671 (83%)	0.005
NRP used	21 (34.4%)	2861 (9.2%)	<0.001
Biopsy obtained	61 (100%)	20 255 (65.4%)	<0.001
DGF	21 (34%)	13 709 (44%)	0.2
PNF	1 (1.6%)	573 (1.9%)	>0.9
Treatment for rejection within 6 mo	1 (1.6%)	945 (3.1%)	>0.9

Data presented as median (IQR) or n (%).

^*a*^Wilcoxon rank-sum test; Pearson’s chi-square test; Fisher exact test.

BMI, body mass index; DCD, donation after circulatory death; DGF, delayed graft function; EPTS, estimated posttransplant survival; es-MP, ex situ machine perfusion; IQR, interquartile range; KDPI, Kidney Donor Profile Index; NRP, normothermic regional perfusion; PNF, primary nonfunction.

### Characteristics of Liver Donors and Recipients

Recipients of livers from septuagenarian donors were significantly older (66.5 versus 59.0 y, *P* < 0.001) than liver recipients in the non-septuagenarian cohort (Table 3). Among transplanted livers, there was no significant difference in macrosteatosis between cohorts (5 versus 5%, *P* = 0.8), whereas DRI scores were significantly higher (2.98 versus 2.01, *P *< 0.001). Recipients of septuagenarian livers had significantly lower allocation and laboratory MELD scores at transplant (15 versus 21 *P* < 0.001; 16 versus 19, *P* < 0.001). Transplanted livers from septuagenarian donors were significantly more likely to have undergone es-MP (74.0 versus 53.2%, *P* = 0.003). They were also significantly more likely to originate from a donor where NRP was used (56.0 versus 17.5%, *P* < 0.001). All recipients of livers from septuagenarian donors received whole liver grafts. Among the non-septuagenarian graft recipients, 2 (<0.1%) received a split liver. A single recipient of a septuagenarian graft (2.0%) received a simultaneous liver-kidney transplant, compared with 549 (7.7%) in the non-septuagenarian recipients.

**TABLE 3.** Demographic and clinical characteristics of liver recipients

Characteristic	Septuagenarian DCD (N = 50)	Non-septuagenarian DCD (N = 7135)	*P* ^*a*^
Recipient			
Age, y	66 (61–69)	59 (51–65)	<0.001
Sex, male	21 (42.0%)	4692 (65.7%)	<0.001
BMI, kg/m^2^	29.1 (25.3–31.8)	28.5 (25.0–32.8)	0.6
Cause of liver disease			0.5
Acute liver failure	0 (0%)	50 (0.7%)	
Alcohol-associated cirrhosis	14 (28.0%)	2206 (30.9%)	
Alcohol-associated hepatitis	0 (0%)	175 (2.5%)	
Cholestatic disease	1 (2.0%)	278 (3.9%)	
HCC	9 (18.0%)	1400 (19.6%)	
HCV	0 (0%)	301 (4.2%)	
MASH	17 (34.0%)	1490 (20.8%)	
Other/unknown	9 (18.0%)	1235 (17.3%)	
Waitlist time, d	90 (25–241)	78 (20–229)	0.9
Laboratory MELD score	16 (11–19)	19 (14–24)	<0.001
Allocation MELD score	15 (13–19)	21 (16–25)	<0.001
Exception case	13 (26.0%)	1926 (26.9%)	0.9
Retransplant	0 (0%)	81 (1.1%)	>0.9
Multiorgan transplant			0.3
Kidney	1 (2.0%)	549 (7.7%)	
Heart	0 (0%)	15 (0.2%)	
Dialysis before transplant	0 (0%)	460 (6.4%)	0.14
Ventilated before transplant	0 (0%)	45 (0.6%)	>0.9
Donor			
Age, y	71 (70–73)	45 (33–55)	<0.001
BMI, kg/m^2^	27 (24–33)	28 (24–32)	0.9
Terminal ALT, U/L	32 (19–58)	38 (22–79)	0.5
Terminal AST, U/L	47 (28–73)	48 (30–79)	0.5
Terminal bilirubin, mg/dL	0.55 (0.40–0.80)	0.50 (0.30–0.80)	0.2
History of diabetes	10 (20.0%)	996 (13.9%)	0.2
History of hypertension	32 (64.0%)	2690 (37.7%)	<0.001
Transplant			
Graft type			>0.9
Whole	50 (100%)	7132 (99.9%)	
Split	0 (0%)	3 (<0.1%)	
Preservation time, h	13.4 (5.0–19.5)	8.6 (5.2–16.6)	0.069
Distance from transplant center, nautical miles	151 (25–384)	91 (17–204)	0.10
es-MP used	37 (74.0%)	3800 (53.2%)	0.003
NRP used	28 (56.0%)	1250 (17.5%)	<0.001
Biopsy obtained	26 (52.0%)	2913 (40.8%)	0.11
Liver macrosteatosis, %	5 (0–10)	5 (0–10)	0.8
DRI, score	2.98 (2.66–3.37)	2.01 (1.70–2.41)	<0.001
PNF	0 (0%)	74 (1.0%)	0.8
Treated for rejection within 6 mo	4 (8.0%)	435 (6.1%)	0.5

Data presented as median (IQR) or n (%).

^*a*^Wilcoxon rank-sum test; Pearson’s chi-square test; Fisher exact test.

ALT, alanine transaminase; AST, aspartate aminotransferase; BMI, body mass index; DCD, donation after circulatory death; DRI, donor risk index; es-MP, ex situ machine perfusion; HCC, hepatocellular carcinoma; HCV, hepatitis C virus; IQR, interquartile range; MASH, metabolic dysfunction-associated steatohepatitis; MELD, model for end-stage liver disease; NRP, normothermic regional perfusion; PNF, primary nonfunction.

### Characteristics of Lung Donors and Recipients

Of the 3 recipients of 6 lung grafts (all dual grafts) from septuagenarian donors, only 2 were available for further analysis, as the remaining recipient underwent transplant outside of the United States. There were 1264 recipients of non-septuagenarian DCD lung grafts, of whom 1068 received dual grafts (84.4%). Of recipients with available data, the recipients of septuagenarian grafts had a median age of 68.0 y as compared with 63.0 y for recipients of non-septuagenarian grafts. One (50%) of the transplanted septuagenarian lungs underwent es-MP, compared with 329 (26%) in the non-septuagenarian cohort.

### Posttransplant Outcomes by Cohort

In the kidney recipient cohort, there was no significant difference in the 1-y all-cause graft survival probability between the cohorts (92.9 versus 91.6%, *P* = 0.99; Figure 2A). No significant difference in the rate of DGF (34.4 versus 44.3%, *P* = 0.2) or PNF (1.6 versus 5.0%, *P* = 0.4) between cohorts was observed. The rate of rejection requiring treatment at 6 mo was not significantly different between cohorts (1.6 versus 3.1%, *P *> 0.9).

**FIGURE 2.**
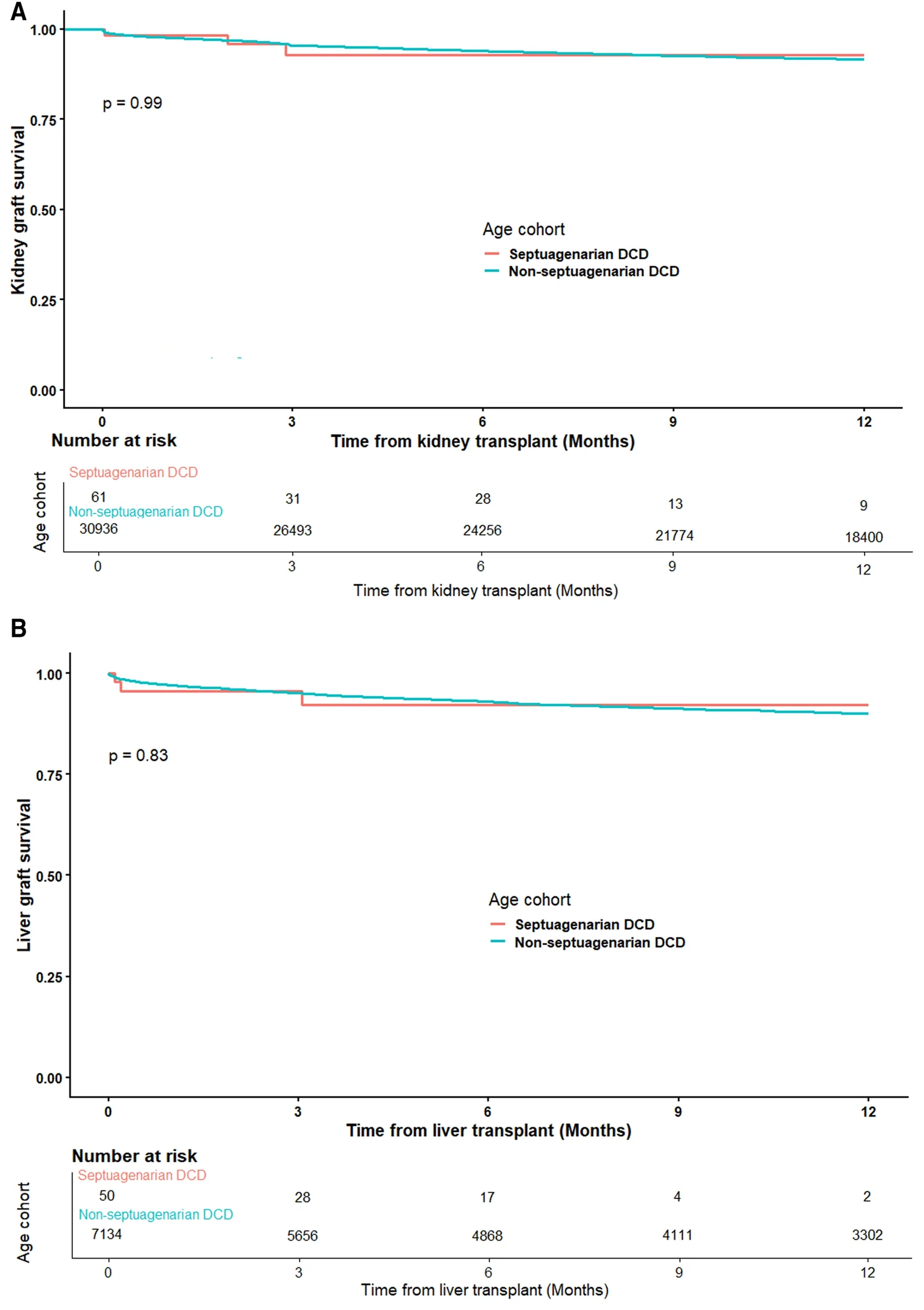
Kaplan-Meier graft survival curves after kidney and liver transplantation. A, One-year graft survival after kidney transplantation between septuagenarian and non-septuagenarian cohorts. B, One-year graft survival after liver transplantation between septuagenarian and non-septuagenarian cohorts. DCD, donation after circulatory death.

In the liver recipient cohort, there was no statistically significant difference in 1-y all-cause graft survival probability between cohorts (92.3 versus 90.1%, *P* = 0.82; Figure 2B). The rate of PNF did not vary significantly between the cohorts (0.0 versus 1.0%, *P* = 0.8), nor did the rate of rejection requiring treatment at 6 mo (8.0 versus 6.1%, *P *= 0.5). Of liver recipients with graft failure, there was not a significant difference in the prevalence of ischemic cholangiopathy (0.0 versus 24.3%, *P *> 0.9).

Out of 2 lung recipients of septuagenarian grafts with available data, 1 was alive, and 1 had died 120 d after transplant from multiple organ failure. There were no instances of primary nonfunction among the lung recipients of septuagenarian grafts, compared with 1.4% of non-septuagenarian graft recipients (*P* > 0.9).

### Geographic Analysis

Stratification of septuagenarian donors by geographic region demonstrated that the largest recovery volume occurred in the Midwest (OPTN Region 10) followed by New England (OPTN Region 1) and the Southwest (OPTN Region 5), although varying recovery volumes were observed across the nation (Figure 3A). Analysis of total numbers of septuagenarian DCD donors recovered by OPO during the study period demonstrated that the majority originated from 6 aggressive OPOs, with 2 OPOs recovering >30 DCD septuagenarians each (33.8% of national cases; Figure 3B). Almost half (26/55; 47.2%) of OPOs did not recover any septuagenarian DCD donors during the study period. OPO use of NRP demonstrated a moderate and significant correlation with the number of septuagenarian DCD donors recovered (*R* = 0.3, *P *= 0.022; **Figure S1, SDC,** https://links.lww.com/TXD/A910).

**FIGURE 3.**
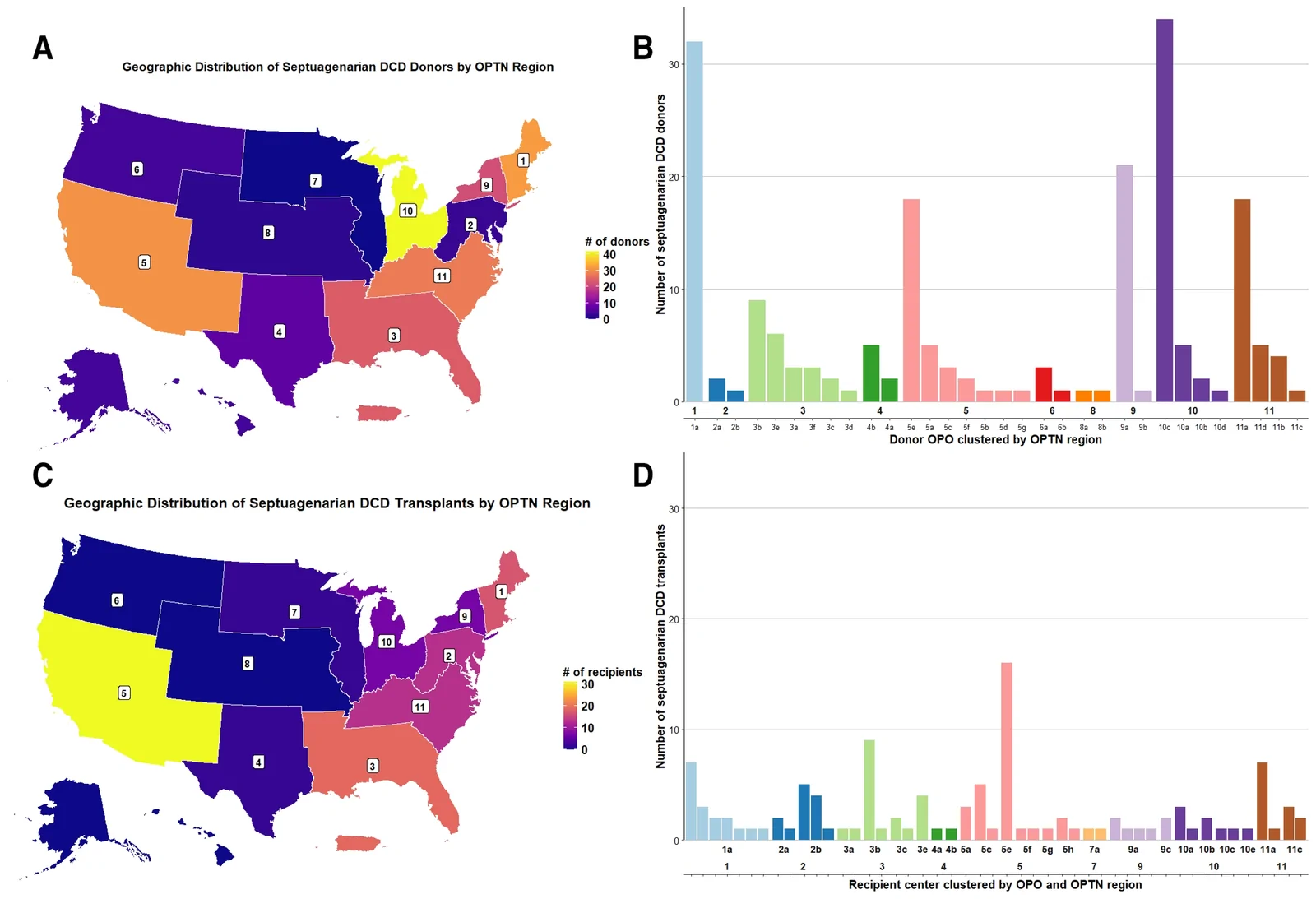
Geographic distribution of septuagenarian DCD donors and transplants. A, Geographic distribution of septuagenarian DCD donors recovered by OPTN region. B, Number of septuagenarian DCD donors by OPO. C, Geographic distribution of transplants using septuagenarian DCD donors by OPTN region. D, Number of transplants using septuagenarian DCD donors among transplant centers. DCD, donation after circulatory death; OPO, organ procurement organization; OPTN, Organ Procurement and Transplantation Network.

Geographic distribution of transplanted septuagenarian DCD grafts also demonstrated significant variation, with the majority of recipients in the Southwest (OPTN Region 5) and the South (OPTN Region 3), followed by New England (OPTN Region 1; Figure 3C). Utilization of septuagenarian DCD grafts per transplant center (classified by OPO-level) demonstrated that 4 (1.8%) of 218 centers transplanted 34.8% of all recipients of septuagenarian grafts in the study period (Figure 3D). Of the 47 centers which used septuagenarian DCD grafts, the majority (55.3%) used only a single graft.

The median proportion of septuagenarians of all DCD donors recovered among the aggressive OPOs was 6.5%. Using the data from 2024, if all OPOs had increased septuagenarian DCD utilization to that of the aggressive group, this would have resulted in the recovery of 351 additional donors annually.

## DISCUSSION

To address the ongoing organ shortage in the context of global aging, strategies to pursue donors of advanced age are critical.^5^ With the number of individuals older than 70 y projected to more than double globally by 2050,^31^ the potential septuagenarian donor pool will only increase. In this study, we present the first analysis using national data on septuagenarian DCD utilization in the United States. The study found a significant shift in the recovery of septuagenarian DCD donors: from 3 in 2020 to 141 in 2024. It is noteworthy that there was no discernible difference in 1-y graft survival after kidney or liver transplant, and PNF rates of <2.0% across all organs. Despite these encouraging outcomes, there has been significant variation in septuagenarian DCD recovery across OPOs. Two OPOs (3.6%) of 55 nationally recovered 33.8% of all the septuagenarian DCD donors in this study, whereas 26 (47.2%) recovered none. If all OPOs nationally were to pursue septuagenarian DCDs to the extent of the aggressive OPOs, 351 more annual donors could have been added. Given the ongoing organ shortage and aging population, septuagenarian DCD donors should be considered for transplantation.

Significantly lower recovery rates in the septuagenarian cohort may reflect concerns surrounding DCD grafts from older donors and the perception of significantly poorer outcomes predicted by prior studies.^10,11^ A recent survey of DCD liver transplant centers reported that 72% (18/25) of centers use an age cutoff of 70 y or less, inherently limiting the usability of these grafts.^32^ The results of prior work, however, suggest that strict age cutoffs for DCD acceptance may be unfounded, as analysis of transplantation with septuagenarian donors has found no worsened outcomes with properly selected donors/recipients in kidney,^33^ liver,^34^ and lung^18^ transplant. In concordance with these findings, this study found no decrease in 1-y graft survival among recipients of septuagenarian DCD kidney (92.9 versus 91.6%) or liver grafts (92.3 versus 90.1%). Furthermore, no increase in PNF or rejection rates across all organs were observed, and DGF rates were likewise equivalent between recipients of septuagenarian and non-septuagenarian kidneys. In the broader context of significantly improving DCD outcomes across organs,^13-15^ and an aging population, donor age alone should not be an exclusion from DCD candidacy.

The favorable posttransplant outcomes in the septuagenarian cohort demonstrated in this study were likely influenced by careful donor and recipient selection and the use of perfusion technology. First, although used kidney grafts were predicted to be high risk with a median kidney donor profile index of 91 (versus 55 in the non-septuagenarian cohort), BMI, prevalence of diabetes, and terminal serum creatinine were equivalent between cohorts, likely representing selection of only the healthiest septuagenarian donors. Similarly, the equivalence of BMI, prevalence of diabetes, liver enzymes, and serum bilirubin between cohorts likely represents increased selectivity among septuagenarian liver donors. Second, donor/recipient matching is known to be crucial for success in marginal donor transplantation.^35,36^ Recipients of septuagenarian DCD kidneys had markedly higher estimated posttransplant survival scores than non-septuagenarian recipients (76 versus 57), reflecting selection of recipients with shorter times needed for graft function. At present, septuagenarian DCD kidney grafts would likely be best used for older, high-EPTS candidates (eg, EPTS >60), as well as those with shorter times on dialysis. Similarly, recipients of septuagenarian DCD livers were significantly older, likely reflecting donor-recipient age matching.^37^ Liver recipients also demonstrated low medical acuity with significantly lower MELD scores and no pretransplant dialysis or ventilation, which has been shown to effectively mitigate the risk of using older grafts.^38^ Finally, the use of perfusion technology, both es-MP and NRP, was extensive in the septuagenarian cohort, with 74.0% and 56.0% of transplanted septuagenarian livers undergoing es-MP and NRP, respectively. With the restoration of warm, oxygenated perfusion, the use of this technology likely reduced ischemic injury of the older grafts and contributed to the observed favorable outcomes, and is likely advisable when considering septuagenarian DCD transplantation.^22,39^

Analysis of both OPO-level pursuit of and transplant center utilization of septuagenarian DCD donors demonstrated significant heterogeneity. Prior work has established significant geographic variation in the use of older^37^ as well as DCD grafts,^39^ with a small group of aggressive centers driving national trends. Similarly, in this study, recovery of septuagenarian DCD donors was concentrated among a small group of OPOs. Conversely, the grafts were distributed to a wider pool of transplant centers, many of which used only a single septuagenarian DCD graft during the study period. Although 1 center-OPO pair in region 5 demonstrated very strong concordance between recovery and utilization, the pattern implies that aggressive OPOs are pursuing septuagenarian DCD donors “independently” of their local transplant centers and providing the grafts regionally or nationally instead. Given the strong correlation between perfusion technology use and septuagenarian DCD recovery, these aggressive OPOs are likely able to leverage perfusion to (1) provide sufficient organ diagnostics and assurances of quality to gain acceptance from a distant center and (2) preserve the organs with es-MP as to logistically enable delivery of the graft. As age cutoffs for center acceptance of DCD grafts continue to increase,^40^ it is expected that the opportunities to utilize septuagenarian DCD donors will increase significantly. OPOs that currently do not recover septuagenarian DCD donors should be aware that grafts from these donors are transplantable and show favorable outcomes when recovered using perfusion technology. Septuagenarian DCD donors may be of increasing relevance as the regulatory landscape of organ transplantation in the United States continues to evolve. The change in OPO Conditions for Coverage in December of 2020 was notable for instituting objective criteria for “eligible deaths,” including for individuals aged up to 75 y.^41^ Similarly, the OPTN Modernization Act passed in September of 2023 was explicitly intended to increase competition among OPOs.^42^ These regulatory changes may have partly motivated the changes in practice observed in this study and are likely to continue to influence pursuit of DCD in septuagenarians.

This study is limited by its design as a retrospective analysis of national registry data. The UNOS Standard Transplant Analysis and Research database only contains deceased donors who were ultimately taken for organ recovery. Potential donors who were considered but declined for recovery are not included, limiting the full assessment of utilization patterns.^19^ The utilization of NRP is also not officially recorded in the database, and the utilization of liver es-MP is likely to be significantly underreported.^28^ The inferences used to assess NRP and es-MP utilization create the possibility of inaccurately classified cases. Given the substantial impact of these technologies, improved data collection is critical to support the ongoing utilization of older grafts. The study is also limited by follow-up time, due to the significant increase in the number of septuagenarian DCD donors in 2024. Although the low rates of PNF and favorable short-term graft survival support the aggressive utilization of septuagenarian DCD donors, further longitudinal studies are needed to evaluate long-term outcomes.

In conclusion, the utilization of septuagenarian DCD grafts has increased dramatically in the United States, which may be largely attributable to the adoption of NRP and es-MP. Graft survival from septuagenarian DCD donors was comparable with that of non-septuagenarian DCD grafts in kidney and liver transplantation, which could be driven by low-risk donor selection and donor/recipient matching based on age, medical acuity, and anticipated graft lifespan. Given that only 2 OPOs (of 55) performed 33.8% of septuagenarian DCD recoveries, more OPOs should consider the recovery of septuagenarian DCD donors, especially in conjunction with perfusion technologies.

## Supplementary Material
