# Supplementary material for: Utilization and Outcomes of Septuagenarian Donation After Circulatory Death Donors
Source: Transplant Direct. 2026 Sep 25;12(10):e2014. doi: 10.1097/TXD.0000000000002014 (PMC13623291; doi:10.1097/TXD.0000000000002014)
Supplement: Supplementary file 1 [file txd-12-e2014-s001.pdf]

SDC, Figure 1: (A) Correlation between number of donors utilizing NRP and number of septuagenarian DCD donors per OPO. (B) Correlation between number of donors utilizing liver es-MP and number of septuagenarian DCD donors per OPO. (C) Correlation between number of donors utilizing kidney es-MP and number of septuagenarian DCD donors per OPO. (D) Correlation between number of donors utilizing lung es-MP and number of septuagenarian DCD donors per OPO.

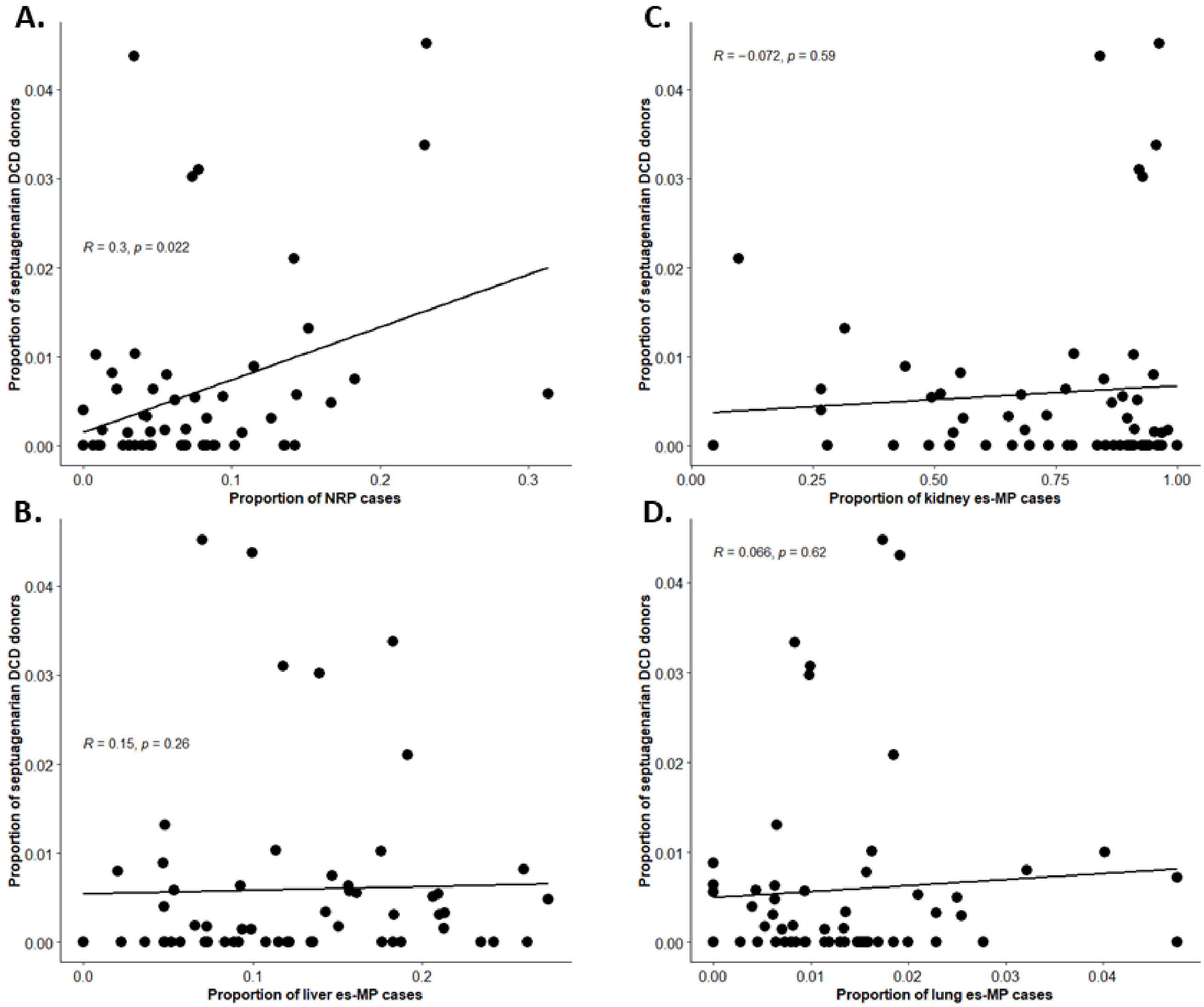

SDC, Table 1: Reasons for kidney non-utilization for septuagenarian and non-septuagenarian DCD organ donors

Reason	Septuagenarian DCD N = 296[1]	Non-septuagenarian DCD N =15,600[1]	*P* value[2]
**Donor factors**			<0.001
Biopsy findings	32 (10.8%)	2070 (13.2%)	
Poor organ function	1 (<1.0%)	486 (3.1%)	
Donor medical history	36 (12.1%)	273 (1.7%)	
Donor social history	1 (<1.0%)	4 (<1.0%)	
Diseased organ	2 (<1.0%)	242 (1.5%)	
Anatomic abnormalities	5 (<1.0%)	429 (2.7%)	
Inadequate urine output	0 (0%)	6 (<1.0%)	
Trauma to organ	0 (0%)	78 (<1.0%)	
Trauma to vasculature	1 (<1.0%)	90 (<1.0%)	
Ureteral damage	0 (0%)	37 (<1.0%)	
Organ not as described	0 (0%)	42 (<1.0%)	
Infection/CMV+/Hepatitis+	0 (0%)	60 (<1.0%)	
Too old on ice	0 (0%)	125 (<1.0%)	
Too old on pump	0 (0%)	46 (<1.0%)	
**Recipient/Preservation factors**			>0.9
No recipient located	187 (63.1%)	9126 (58.5%)	
Warm ischemic time too long	2 (<1.0%)	171 (1.0%)	
Recipient unsuitable	0 (0%)	36 (<1.0%)	
**Other/Unknown**	49 (16.5%)	5361 (34.3%)	<0.001

[1] n (%)

[2] Fisher's exact test

SDC Table 2: Reasons for liver non-utilization for septuagenarian and non-septuagenarian DCD organ donors

**Reason**	**Septuagenarian DCD N = 8**[1]	**Non-septuagenarian DCD N =2,321**[1]	***P* value**[2]
**Donor factors**			0.2
Biopsy findings	1 (12.5%)	412 (17.7%)	
Poor organ function	0 (0%)	167 (7.2%)	
Donor medical history	0 (0%)	27 (1.1%)	
Diseased organ	2 (25.0%)	115 (4.9%)	
Anatomic abnormalities	0 (0%)	100 (4.3%)	
Trauma to organ	0 (0%)	24 (1.0%)	
Trauma to vasculature	0 (0%)	15 (<1.0%)	
Organ not as described	1 (12.5%)	18 (<1.0%)	
Infection/CMV+/Hepatitis+	0 (0%)	10 (<1.0%)	
Too old on ice	0 (0%)	20 (<1.0%)	
Too old on pump	0 (0%)	3 (<1.0%)	
**Recipient/Preservation factors**			0.4
No recipient located	1 (12.5%)	206 (8.8%)	
Warm ischemic time too long	0 (0%)	409 (17.6%)	
Recipient unsuitable	0 (0%)	24 (1.0%)	
**Other/Unknown**	3 (37.5%)	770 (33.1%)	0.06

[1] n (%)

[2] Fisher's exact test
